# High calcium concentration in bones promotes bone metastasis in renal cell carcinomas expressing calcium-sensing receptor

**DOI:** 10.1186/1476-4598-13-42

**Published:** 2014-02-28

**Authors:** Elke Joeckel, Tobias Haber, Dirk Prawitt, Kerstin Junker, Christian Hampel, Joachim W Thüroff, Frederik C Roos, Walburgis Brenner

**Affiliations:** 1 Department of Urology, Johannes Gutenberg University Medical Center, Langenbeckstr 1, Mainz 55131, Germany; 2 Department of Pediatrics, Johannes Gutenberg University Medical Center, Langenbeckstr 1, Mainz 55131, Germany; 3 Department of Urology, Saarland University, Kirrberger Str. 100, Homburg/Saar 66421, Germany; 4 For the German Renal Cell Tumor Network, Homburg/Saar, Germany

**Keywords:** Renal cell carcinoma, Bone metastasis, Calcium-sensing receptor, Microenvironment

## Abstract

**Background:**

The prognosis for renal cell carcinoma (RCC) is related to a high rate of metastasis, including 30% of bone metastasis. Characteristic for bone tissue is a high concentration of calcium ions. In this study, we show a promoting effect of an enhanced extracellular calcium concentration on mechanisms of bone metastasis via the calcium-sensing receptor (CaSR) and its downstream signaling molecules.

**Methods:**

Our analyses were performed using 33 (11/category) matched specimens of normal and tumor tissue and 9 (3/category) primary cells derived from RCC patients of the 3 categories: non-metastasized, metastasized into the lung and metastasized into bones during a five-year period after nephrectomy. Expression of CaSR was determined by RT-PCR, Western blot analyses and flow cytometry, respectively. Cells were treated by calcium and the CaSR inhibitor NPS 2143. Cell migration was measured in a Boyden chamber with calcium (10 μM) as chemotaxin and proliferation by BrdU incorporation. The activity of intracellular signaling mediators was quantified by a phospho-kinase array and Western blot.

**Results:**

The expression of CaSR was highest in specimens and cells of patients with bone metastases. Calcium treatment induced an increased migration (19-fold) and proliferation (2.3-fold) exclusively in RCC cells from patients with bone metastases. The CaSR inhibitor NPS 2143 elucidated the role of CaSR on the calcium-dependent effects. After treatment with calcium, the activity of AKT, PLCγ-1, p38α and JNK was clearly enhanced and PTEN expression was almost completely abolished in bone metastasizing RCC cells.

**Conclusions:**

Our results indicate a promoting effect of extracellular calcium on cell migration and proliferation of bone metastasizing RCC cells via highly expressed CaSR and its downstream signaling pathways. Consequently, CaSR may be regarded as a new prognostic marker predicting RCC bone metastasis.

## Background

Approximately 30% of patients with renal cell carcinoma (RCC) develop bone metastases during the course of the disease. The median survival of patients presenting with bone metastases at the time of RCC diagnosis is 10.6 months [1]. Bone metastases from RCC are destructive and cause osteolysis. The consequences are skeletal complications such as bone pain, pathologic fractures, hypercalcaemia and spinal cord and nerve root compression [2,3]. The prognosis for patients is poor because RCC bone metastases are practically insensitive to standard therapy, such as conventional radiation or chemotherapy [4,5].

The formation of metastases is a process involving multiple steps. First, tumor cells escape from the primary tumor and migrate towards the blood vessels. After dissemination by the blood flow they become trapped in small capillaries in the secondary organ. The tumor cells adhere to the endothelium and finally invade through the capillary walls into the subendothelial tissue [6,7]. The formation of metastases depends on the microenvironment of the secondary organ being compatible to the invading tumor cell [8]. The organ specificity of metastasis can be caused by a particular constitution of the endothelium, for example bone marrow sinusoid capillaries being highly fenestrated [9] and/or the chemotactical behavior and tumor growth promoting effect of the subendothelial tissue, including the composition of extracellular matrix compounds and growth factors [10].

The high frequency of bone metastases deriving from RCC indicates an environment in this organ with the ability to promote renal tumor cells with supporting processes such as cell motility, adhesive interactions, cell proliferation and tumor growth. Bone remodeling is a physiological process of permanent bone resorption by osteoclasts and bone formation by osteoblasts. During this process calcium ions are released into the bone matrix in high concentrations [3,11].

The impact of extracellular calcium on cells implicates an activation of the calcium-sensing receptor (CaSR), a G-protein-coupled receptor [12]. It is highly expressed in the healthy kidney [13] and governs several functions: regulation of extracellular calcium concentration and inorganic phosphate homeostasis, mono- and divalent cation transport, acidification and concentration of urine as well as renin release [14-16]. When activated through enhanced extracellular calcium concentration, CaSR coordinates cellular responses via a variety of intracellular signaling pathways. These finally lead to a modulation of cell proliferation, differentiation, migration and apoptosis [17]. In breast cancer, the expression of CaSR correlates with the formation of bone metastases [18].

Since CaSR is highly expressed in epithelial cells of the healthy kidney, we also assume a relatively high expression of this receptor in renal tumor cells and a promoting effect of calcium on bone metastatic processes, which has not been studied in detail. In this study we investigated the oncogenic properties of CaSR in RCC and the influence of extracellular calcium on the formation of RCC bone metastases. We correlated *CaSR* mRNA expression in primary RCC tissue samples with the localization of metastases. Additionally, the expression of CaSR was analyzed in primary RCC cells of patients with different metastatic localizations. To study the effect of extracellular calcium on metastatic behavior, we quantified the chemotactical migration and cell proliferation of these RCC cells under calcium influence. The molecular mechanisms responsible for the effects observed were analyzed by quantifying the activity of intracellular signaling pathways, especially the AKT and MAPK pathways and its regulatory phosphatase PTEN. The elucidation of the importance of calcium and CaSR in the process of bone metastasis could reveal new prognostic markers and contribute to the development of new target therapies.

## Results

### Tissue specimens of RCC patients developing bone metastases show a high *CaSR* expression

**Figure 1:**
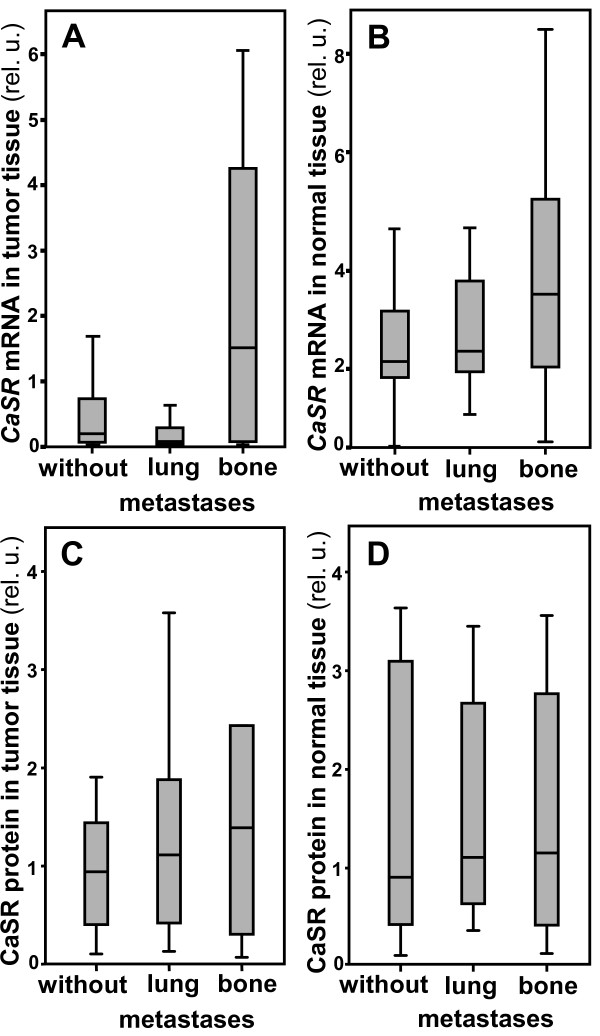
***CaSR *expression in renal tissue specimens of RCC patients with varying metastatic sites.** Total RNA was isolated and reverse transcribed from renal tumor **(A)** and normal kidney **(B)** tissue specimens from 33 patients with RCC developing no metastases, lung or bone metastases within 5 years after nephrectomy (11/category). *CaSR* mRNA was quantified by real time PCR. Real time PCR of TBP was performed simultaneously for reference. Values are demonstrated as relative units (rel. u.) *CaSR* was highly expressed in normal kidney tissue and in renal tumor tissue of patients who developed bone metastases within 5 years after nephrectomy. In renal tumor tissue of patients with no or with lung metastases almost no *CaSR* was detectable. From the same tissue specimens protein was extracted and CaSR was quantified by Western blot. A similar trend was observed, although the effect was even less pronounced **(C** and **D)**. Box plots show medians (central lane), 25% and 75% percentiles (lower and upper side of the box) and minimum and maximum (lower and upper bars). Outliers are not shown.

Quantification of the CaSR expression in RCC was performed by analyzing tumor and normal tissue specimens from RCC patients without metastases and from patients developing lung or bone metastases within 5 years after nephrectomy (11 patients/category) by quantitative RT-PCR. The results were correlated with the localization of the metastatic sites. In tumor specimens of patients developing bone metastases, *CaSR* mRNA expression was 7.9-fold higher than in tumor specimens of patients without metastases (Figure 1A). Tumor specimens from patients with no metastases or with lung metastases expressed *CaSR* mRNA moderately. In normal renal tissue, *CaSR* expression was considerably higher than in tumor specimens. In normal renal tissue of patients developing bone metastases, *CaSR* mRNA expression was 1.8-fold higher than in specimens of patients without metastases (Figure 1B). Analyzing the CaSR protein in the tissue specimens we observed a similar trend, although the effect was even less pronounced (Figure 1C and D).

### Bone metastatic primary RCC cells show a high CaSR expression

**Figure 2:**
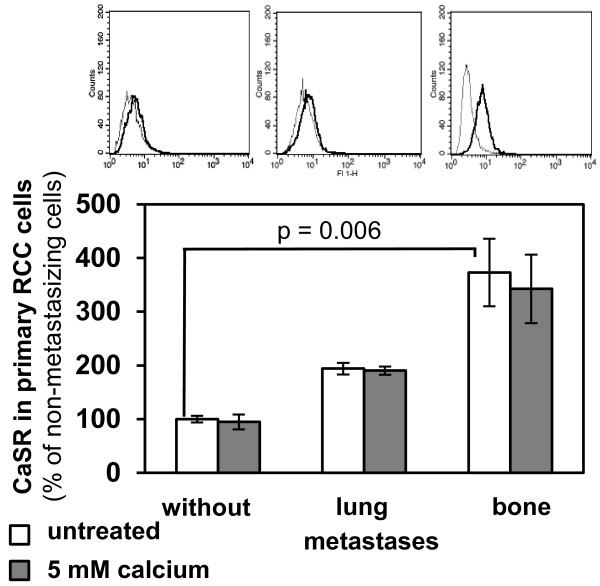
**CaSR expression in primary RCC cells of different metastatic potential.** CaSR expression was quantified in primary RCC cells of 9 patients developing no metastases, lung or bone metastases within 5 years after nephrectomy (3/category) after treatment with 5 mM calcium for 30 min by flow cytometry. Exemplarily demonstrated in the histograms are IgG control (gray line) and calcium treated cells (black line). CaSR expression was highest in patients with bone metastases, p = 0.006. Treatment with calcium (5 mM) had no effect on CaSR expression. The columns represent mean values of 10.000 counted cells and standard error in% of values of non-metastasizing tumor cells (= cells of patients without metastases). An isotype specific IgG control antibody was used for control.

The expression of CaSR in primary RCC cells was determined by flow cytometry. Corresponding to the results obtained from tissue specimens, CaSR expression in RCC cells cultivated from patients developing bone metastases was 3.7-fold higher than in cells from patients without metastases (p = 0.006). In cells from patients developing lung metastases, CaSR expression was 1.9-fold higher than in non-metastasizing RCC cells. Treatment with 5 mM calcium had no influence on CaSR expression of RCC cells (Figure 2).

### Extracellular calcium stimulates migration and proliferation of bone metastasizing primary RCC cells

**Figure 3:**
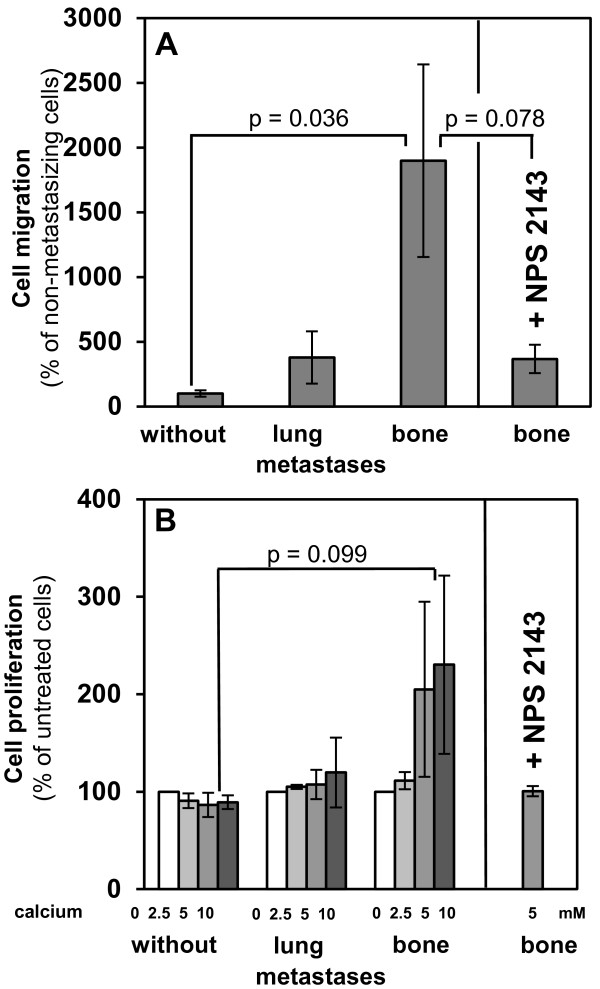
**Calcium dependent cell migration and proliferation of RCC cells of different metastatic potential. A**, migration of primary RCC cells of 9 patients developing no metastases, lung or bone metastases within 5 years after nephrectomy (3/category) was determined in a Boyden chemotaxis chamber using calcium (10 mM) as chemotactic agent. Migration of RCC cells from patients with bone metastases was clearly increased, p = 0.036. The columns represent mean values and standard error in% of values from non-metastasizing cells (= without metastases). **B**, primary RCC cells of 9 patients developing no metastases, lung or bone metastases within 5 years after nephrectomy (3/category) were treated with calcium of different concentrations for 30 min. Proliferation of cells from patients with bone metastases, determined by BrdU incorporation, was increased after treatment with calcium, p = 0.099. The columns represent mean values and standard error in% of untreated cells. **A**, **B**, using the allosteric CaSR inhibitor NPS 2143 (5 μM) bone metastatic RCC cells were no longer responsive to calcium.

Since the CaSR expression was enhanced in tumor tissue and primary cells from patients who developed bone metastases, we investigated the influence of extracellular calcium in processes of metastasis. The migratory potential of primary RCC cells was analyzed in a Boyden chemotaxis chamber using calcium as chemotaxin. To investigate the influence of calcium on proliferation of these primary RCC cells, they were incubated with calcium for 30 min and cell proliferation was determined by BrdU incorporation. The migratory potential of RCC cells from patients with bone metastases was clearly increased (19-fold, p = 0.036) compared to non-metastasizing cells. Cells from patients with lung metastases also had a higher migratory potential than non-metastasizing cells (3.8-fold). Thus, in contrast to metastasizing cells, non-metastasizing cells were only slightly responsive to calcium as a chemotaxin (Figure 3A). Furthermore, in bone metastatic RCC cells extracellular calcium increased proliferation in a concentration-dependent manner up to 2.3-fold. RCC cells from patients with no metastases or with lung metastases were not influenced by elevated calcium concentrations (Figure 3B). Using the allosteric CaSR inhibitor NPS 2143, bone metastatic RCC cells were no longer responsive to calcium (cell migration: p = 0.078, proliferation p = 0.154), which confirmed the effect of calcium via the CaSR (Figure 3A, B). These results show that elevated extracellular calcium promotes CaSR dependent migration and proliferation of primary RCC cells with a high potential for building skeletal metastases.

### Extracellular calcium enhances the activity of AKT, PLCγ-1, JNK, p38α, paxillin and reduces the expression of PTEN

**Figure 4:**
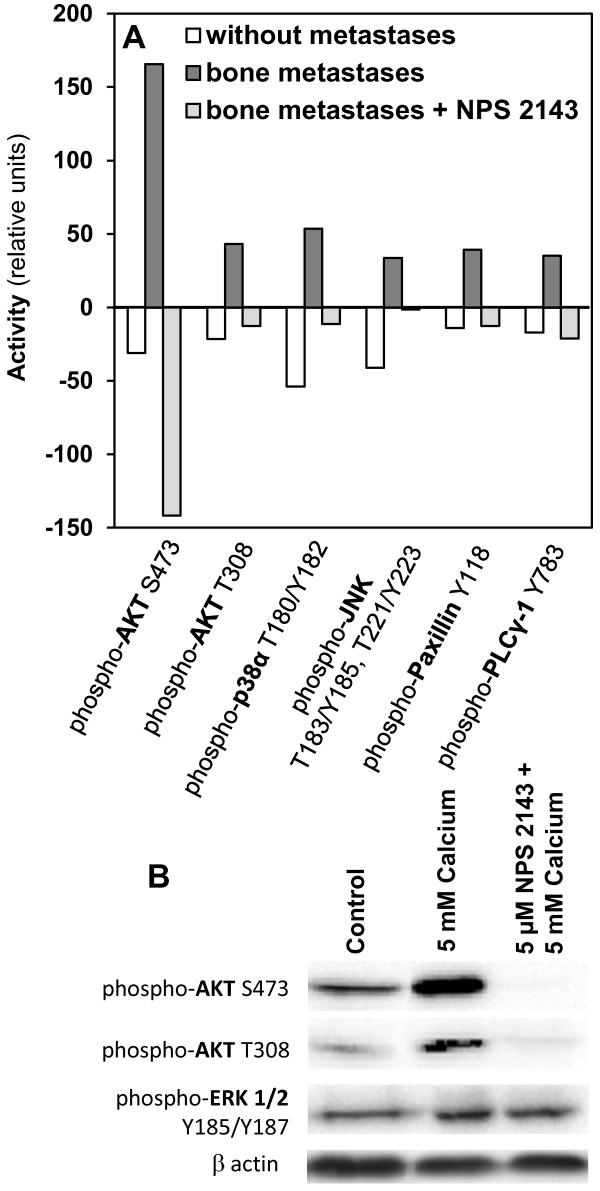
**Activity of intracellular kinases in primary RCC cells of different metastatic potential after calcium treatment. (A)** Activity of AKT, p38α, JNK, paxillin and PLCγ-1 was analyzed by measuring the phosphorylation status in primary RCC cells of patients developing no metastases or bone metastases within 5 years after nephrectomy via a human phospho-kinase array. Values of calcium treated cells (5 mM, 30 min) with or without pretreatment with NPS 2143 were related to untreated cells. The activity of AKT, PLCγ-1, p38α, JNK and paxillin was increased in bone metastasizing cells after treatment with calcium. This effect was inhibited using NPS 2143. The columns represent the amount of antigen determined by computer-aided integration of the bands after subtraction of the background. Demonstrated are the mean values of each lysate in% of untreated non-metastasizing cells (= without metastases). **(B)** Western blot analysis of phosphorylated AKT and ERK in cell lysates of untreated, calcium treated (5 mM, 30 min) or NPS 2143 (5 μM, 30 min) following by NPS 2143 + calcium (30 min) treated cells, 100 μg per lane. β-actin served as an internal control.

**Figure 5:**
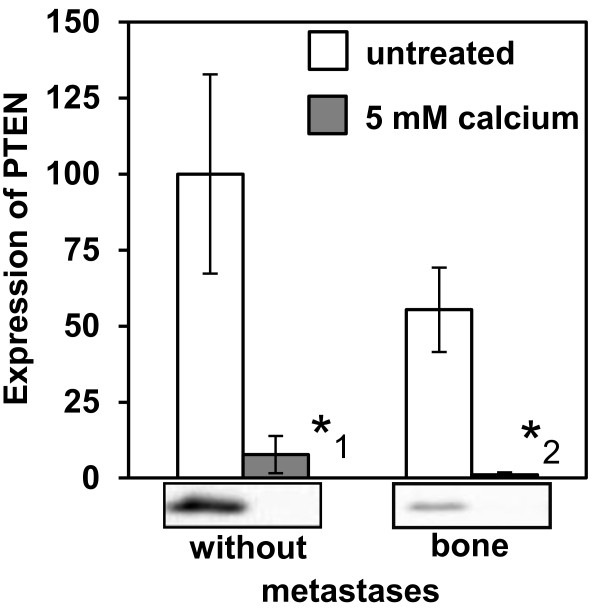
**Expression of PTEN in primary RCC cells of different metastatic potential after treatment with calcium.** Expression of PTEN in primary RCC cells of patients developing no metastases or bone metastases within 5 years after nephrectomy (3/category) was analyzed by Western blotting after treatment with 5 mM calcium for 30 min or without treatment. β-actin was used as a loading control. The expression of PTEN was lower in bone metastasizing cells and further diminished after treatment with calcium. *_1_ p = 0.025, *_2_ p = 0.009. The columns represent the amount of antigen determined by computer-aided integration of the bands after subtraction of the background and referred to the value of β-actin. Demonstrated are the mean values in% of untreated non-metastasizing cells (= without metastases) and the standard error.

To analyze the signaling pathways involved in the calcium-dependent effects demonstrated in this study, we performed a human phospho-kinase array including 46 intracellular kinases. The activity of the kinases was measured by detecting the expression of the phosphorylated molecules. In bone metastasizing cells, the following molecules showed a prominently enhanced phosphorylation status due to their activation by calcium treatment (Figure 4A): AKT (phosphorylation site Ser473: increase of 165%, phosphorylation site Thr308: increase of 43%), PLCγ-1 (increase of 35%), p38α (increase of 53%), JNK (increase of 33%) and paxillin (increase of 39%). In case of NPS 2143 treatment 30 min before adding Calcium, these effects were inhibited. The expression of AKT Ser473 was clearly reduced when cells were NPS 2143 treated. In contrast, ERK was not influenced after calcium treatment of the bone metastasizing cells (data not shown). In non-metastasizing cells, calcium had no activating effect on the analyzed kinases (Figure 4A). Since these kinases are members of the AKT signaling pathway and because the AKT and ERK pathways are mainly activated by CaSR, these results were substantiated by Western blot analysis of phosphorylated AKT and ERK. The results corresponded to those obtained by the human phospho-kinase array (Figure 4B). PTEN expression was markedly reduced in bone metastatic cells to 55%. Calcium treatment resulted in significantly (p < 0.05) reduced PTEN expression in all cell types, in bone metastasizing cells it was almost undetectable (Figure 5).

## Discussion

Although a number of described mechanisms are implicated in the process of cancer metastasis, the organ-selective nature of cancer cells remains poorly understood. The microenvironment of metastatic sites is apparently crucial in several respects e.g. chemotactical power leading tumor cells to a directive migration and a proliferation supporting composition [6-8,19]. This aspect is more important in bones than in other organs, since the highly fenestrated endothelium with no basement membrane implies a weak barrier for tumor cells [9,20].

The inimitable microenvironment in bones implicates a high concentration of calcium since calcium ions are released in the bone matrix in high concentrations during bone turnover [3,21]. Cells have the ability to recognize extracellular calcium by CaSR [12], which in some cancer entities, such as breast cancer, correlates with bone metastasis [18]. In healthy breast tissue, CaSR is responsible for the regulation of calcium concentration in milk and is therefore highly expressed [22]. Healthy kidney tissue also expresses CaSR as a regulator for the resorption of calcium from primary urine [14]. As in breast cancer, renal cancer has a high potential of metastasizing into bones [1], indicating a cancer cell promoting environment in this organ. We investigated the importance of high extracellular calcium concentrations in the determination of bone specificity of RCC metastasis. We analyzed the influence of calcium on cellular behavior and investigated the role of CaSR in processes of metastasis. In tumor tissue specimens of RCC patients with bone metastases during 5 years after nephrectomy, we found a distinctly higher expression of *CaSR*, compared to tumor tissue specimens of patients with no or with lung metastases. This finding implicates the participation of calcium and CaSR in bone metastasis in RCC, which is already constituted in the primary tumor. Interestingly, in the corresponding normal renal tissue of patients with bone metastases, *CaSR* expression was also higher than in the tissue of patients with no or with lung metastases. Therefore the disposition for bone metastasis is possibly already determined in healthy tissue, or alternatively, the primary tumor induces enhanced CaSR in normal renal tissue. These results indicate CaSR being a prognostic marker for the formation of bone metastases in RCC, as also postulated in breast cancer [23,24].

The expression level of CaSR in primary RCC cells showed a pattern similar to that found in tumor tissue. CaSR expression was much higher in cells with a high bone metastatic potential and lower in cells with lung metastatic potential as compared to non-metastasizing cells. In contrast to the expression of CaSR protein in tumor specimens with a 1.5-fold higher value (median) in patients with bone metastases compared to those without metastases, FACS analyses of primary cells showed a significant (p = 0.006) 3.9-fold higher value. This discrepancy may be caused by the fact, that FACS analyses solely detect the biological active CaSR on the cell surface, whereas an analysis of CaSR from a whole protein extract of tissue also detects CaSR additionally stored in vesicles of the cells. The similar tendency in the expression pattern in tumor tissue and RCC cells shows a stability of this attribute during cultivation that advocates further investigation *in vitro* using primary cells. Treatment of RCC cells with calcium had no influence on the expression of CaSR, indicating that calcium can be excluded as a regulator for the expression of CaSR. These results confirm the hypothesis of Rogers et al., who stated that calcium does not regulate the expression of CaSR due to the fact that calcium injected into the inferior vena cava of rats did not significantly change the CaSR expression in the parathyroid gland or in the kidney [25].

Critical steps in metastasis are the migration of tumor cells and cell proliferation in the secondary organ. In this study the influence of calcium on these two steps was analyzed in order to imitate the calcium conditions in the bone microenvironment. In RCC cells metastasizing into bones and expressing a high level of CaSR, the chemotactical potential of calcium was 19-fold higher than in non-metastasizing cells. The CaSR inhibitor NPS 2143 rescinded this effect, evidencing the importance of CaSR in the calcium-dependent reaction. In lung metastasizing RCC cells, calcium-dependent migration was nearly the same as in non-metastasizing cells. This indicates a CaSR-dependent chemotactical attraction of calcium in bones inducing bone metastasis of RCC. Also cell proliferation of bone metastasizing RCC cells, in contrast to non- or lung metastasizing cells, was highly sensitive to calcium, dependent on CaSR. These results indicate a calcium dependence of bone metastasis in RCC, as already defined in the primary tumor by CaSR expression. Since RCC metastasis shows an osteolytic property [2] after initiating bone metastasis, the calcium concentration rises due to bone resorption, which in turn leads to an additional increase of the metastatic potential of RCC cells.

CaSR seems to also play a role in cancer progression of other entities. In bone metastatic breast and prostate cancer cells, calcium and CaSR induces proliferation and motility [26,27]. In parathyroid cancer, CaSR expression reduces Ki67 antigen level and therefore is inversely correlated with cell proliferation [28]. Also in astrocytoma cells [29] and ovarian cells [30,31], CaSR activation induced proliferation and functioned as an oncogene. In contrast to these results, in colon carcinoma cells and neuroblastoma cells, calcium and activation of the CaSR have been shown to inhibit proliferation and induce apoptosis [32,33], indicating CaSR as a tumor suppressor. The impact of calcium and activation of CaSR seem to be dependent on cell type and have to be considered tissue specific.

**Figure 6:**
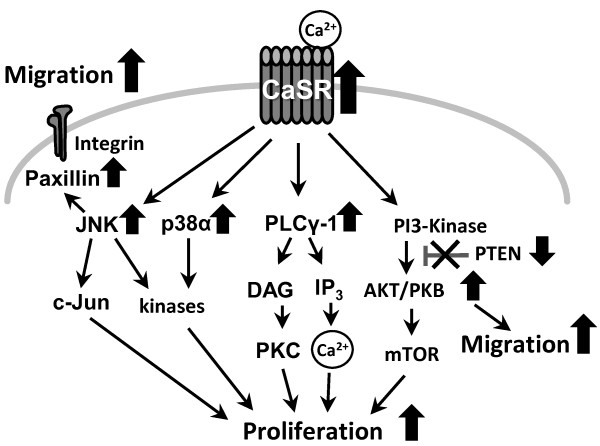
**Signaling pathways involved in the calcium dependent bone metastasis of RCC.** Results obtained in this study are marked with an arrow: arrow upwards means enhancement, arrow downwards means reduction. In cells obtained from patients who developed bone metastases within 5 years after nephrectomy, the expression and activity of CaSR, JNK, paxillin, p38α, PLCγ1 and AKT was enhanced after calcium treatment, PTEN expression was reduced, resulting in an enhanced migration and proliferation.

The CaSR is a G-protein coupled receptor [34] activating several signaling pathways which are known to regulate cell proliferation, differentiation, migration and apoptosis [35,17]. The PI3K (phospatidyl-inositol 3-kinase)/AKT pathway, the PLCγ-1 pathway and the MAPK (mitogen activated protein kinase) cascades are downstream targets of the CaSR [34-36]. In our study, calcium treatment resulted in a clearly enhanced activity of AKT/PKB and PLCγ-1 in bone metastasizing cells but not in non-metastasizing cells. In addition, in bone metastasizing cells, calcium had an activating effect on the MAP kinases p38α and JNK. The focal adhesion adapter protein paxillin as well as c-Jun, both downstream targets of JNK [37,38], showed comparable activity patterns. Inhibiting CaSR with NPS 2143 these enhancements were prevented and the phosphorylation of the signal mediator with the highest calcium sensitivity, AKT, was reduced. The additional reduction of AKT activity after inhibition of CaSR indicates a basement activity of CaSR even without adding calcium. The culture medium contains a low level of calcium not specified by the company. Presumably this low calcium concentration leads to a slightly activation of CaSR and consequently also of AKT-phosphorylation. This effect seems to be inhibited by NPS 2143. The reduced AKT activity induced by NPS 2143 treatment confirms the responsibility of CaSR for the calcium dependent effects. In contrast, calcium had no activating effect on ERK. This suggests AKT, PLCγ-1, p38α and JNK/paxillin signaling pathways, which are described as downstream targets of CaSR [27,36,39,40], being the crucial pathways in the CaSR signaling in RCC cells promoting bone specific metastasis (Figure 6). However, ERK as a downstream target of CaSR is discussed controversially [41] and some studies hypothesize the ERK pathway being involved in extracellular calcium induced cell migration [26,30], again confirming a cell type specific function of CaSR as already described [42]. The main regulator of the AKT pathway is the tumor suppressor PTEN. As an antagonist of the PI3Kinase, PTEN inhibits the activation of AKT and thereby down-regulates cell proliferation and migration [43-45]. In addition, in our former investigations we established a correlation between low PTEN expression in specimens of RCC patients and poor prognosis caused by metastasis [46]. In bone metastasizing RCC cells, PTEN expression was approximately 50% lower than in non-metastasizing cells. The expression of PTEN correlated inversely with the activity of AKT. In addition, the expression of PTEN was highly calcium sensitive. Calcium treatment resulted in an almost complete decline in the expression of PTEN. This implicates that the *per se* low PTEN expression in bone metastasizing RCC cells is further reduced by the bone microenvironment, consequently activating the AKT signaling pathway and promoting bone metastasis (Figure 6).

Our study indicates that bone metastasis of RCC is promoted by an enhanced expression of CaSR. Calcium induces migration and proliferation of bone metastatic RCC cells via CaSR and its signaling pathways and finally promotes bone metastasis. The role of CaSR as a prognostic marker has to be evaluated in further prospective studies.

## Methods

### Specimens

Tissue samples were obtained under sterile conditions from 33 patients with primary RCC who underwent nephrectomy at the Department of Urology, Johannes Gutenberg University Medical Center, Mainz, Germany. The study was performed in agreement with the Declaration of Helsinki and approved by local ethics committee (Landesärztekammer Rheinland-Pfalz, Mainz, Germany: 837.005.09, and the ethics committee of the University Jena, Germany: 2878-07/10). Informed consent was obtained from each patient. Samples from tumor tissue and normal renal cortex, obtained from the opposite kidney pole at a minimum of 3 cm from the tumor, (5x5x5 mm approximately) were shock frozen in liquid nitrogen and stored at −80°C for a period of at least 5 years. The diagnosis of RCC was based on hematoxylin and eosin sections. The development of metastatic sites within 5 years after nephrectomy varied: 11 non-metastasized, 11 metastasized into the lung and 11 metastasized into bones. Tumor specimens were stratified according to histological tumor type, grading, staging, gender, patient’s age and tumor size.

### Quantitative RT–PCR for CaSR mRNA

Total RNA was isolated from renal tissue using a RNA isolation kit (RNeasy Kit, Quiagen, Hilden, Germany). RNA from each tissue was reverse transcribed using a cDNA synthesis kit for RT-PCR (SuperScript II, Invitrogen, Karlsruhe, Germany). cDNA was amplified with a CaSR specific forward primer, 5′-AAG AAA GTT GAG GCG TGG CAG-3′, and a reverse primer, 5′-GAG GTC CCA GTT GAT GAT GGA-3′ (each 10 pmol). CaSR specific amplification was performed in a 10 μl mixture using 5 μl of Light Cycler 480 Cyber Green I Master (Roche) and 1 μl of the cDNA sample. Thermocycling consisted of 50 cycles at 95°C for 5 sec, 61°C for 5 sec and 72°C for 10 sec, followed by a final melting at 95°C. RT-PCR of TBP (TATA-box binding protein) and β-actin from all samples was performed simultaneously for reference, using the arithmetic average of these housekeeping genes.

### Cells and cell culture

Primary RCC cells were isolated from tumor specimens of patients developing bone, lung or no metastases (3 for each category) within 5 years after nephrectomy. Preparation of cells was performed in agreement with the Declaration of Helsinki and approved by local ethics committee (Landesärztekammer Rheinland-Pfalz, Mainz, Germany: 837.005.09, and the ethics committee of the University Jena, Germany: 2878-07/10). Tumor specimens of approximately 1 cm^2^ were obtained from renal tumors shortly after nephrectomy under sterile conditions, separated mechanically with a scalpel and dissociated with 1 mg/ml collagenase II (Sigma, St. Louis, USA) for 30 min at 37°C. To complete dissociation, the samples were pressed through a 70 μm cell strainer. After centrifugation at 1000 rpm for 10 min, the cell pellets were dissolved in AmnioMAX C100 Basal Medium including AmnioMAX C100 Supplement (Gibco, Life Technologies, Darmstadt, Germany). Cells were incubated at 37°C in a humidified atmosphere containing 5% CO_2_ in air. Epithelial origin was proven by immunocytochemical staining of cytokeratins. After growing to semi-confluence, primary cells were cryo-conserved in medium containing 10% DMSO in liquid nitrogen for at least 5 years until usage for the analyses. Cells from patients without-, with lung- or with bone metastases (3 each) were thawed and cultured for 2- to 4- passages. For experimental use and protein extraction, cells were serum-starved for 24 h and treated with 5 mM calcium for 30 min under serum-free conditions. The allosteric CaSR inhibitor NPS 2143 (5 μM) was applied for 1 h (first 30 min without, then 30 min together with calcium). Although NPS 2143 was solved in DMSO resulting in a DMSO concentration in culture medium of 0.005%, we used serum free serum as a control, since we observed an influence of DMSO from a concentration of 0.5% (data not shown).

### Immunocytochemistry

Immunocytochemical staining of cytokeratin pan was performed to prove the epithelial origin of the primary renal tumor cells. Renal tumor cells (5 × 10^3^ cells/ml) were centrifuged on microscope slides and fixed in 100% ethanol for 10 min. Endogene peroxidase was blocked by a 5 min treatment with peroxidase blocking solution (Dako, Hamburg, Germany). Mouse anti-cytokeratin pan monoclonal antibody (Abcam, Cambridge, UK), diluted 1:200 in antibody-diluent (LSAB + −Kit, Dako, Carpinteria, USA), was incubated for 1 h at room temperature. The secondary biotinylated anti-mouse antibody (Dako) was applied for 30 min at room temperature. After using a horseradish peroxidase-conjugated strepatividin-label (LSAB + −Kit, DAKO) for 30 min, cells were treated with DAB (Diaminobenzidine, Dako) for 10 min and counterstained with Mayer’s Hemalm. For all experiments only cytokeratin positive cells were used.

### Flow cytometry

The expression of the CaSR in renal tumor cells was quantified by flow cytometry. Fixation of the cells (2 × 10^6^ cells/ml) was performed in 3.7% paraformaldehyde for 10 min. Mouse monoclonal anti-CaSR (Sigma, St. Louis, USA) was used in a concentration of 0.2 μg/μl, mouse anti-human isotypic control immunglobulines (Dako, Carpinteria, USA) were used in a concentration of 15 μg/μl in PBS containing 1% bovine serum albumin (BSA) for 20 min at 4°C. The secondary alexa flour 488 goat anti-mouse antibody was diluted 1:1000 in 1% BSA/PBS and incubated for 20 min at 4°C in darkness. CaSR expression was quantified in a flow cytometer (BD Calibur, Becton Dickinson, Heidelberg, Germany).

### Cell migration assay

For migration analysis a microchemotaxis chamber (Boyden chamber, Costar, Cambridge, USA) containing an upper and a lower chamber separated by a porous polycarbonate membrane (pore diameter 8 μm; Neuroprobe Inc., Gaithersburg, USA) was employed. The chamber was divided into 48 wells, resulting in an invasion unit with a surface of approximately 7.8 mm^2^. The wells of the lower part of the chamber were coated with 29 μl calcium (10 mM) in serum-free medium or medium alone as control. The lower part was covered with the polycarbonate membrane, previously coated with PBS. 50 μl of the tumor cell suspension (3 × 10^5^ cells/ml) were loaded to the upper part of the chamber in quadruplicate. After an incubation period of 16 h at 37°C in a humidified atmosphere containing 5% CO_2_ in air, cells that did not pass the polycarbonate membrane were removed from the upper side of the porous membrane by washing with a Weise buffer (Merck, Darmstadt, Germany; a potassium dihydrogen phosphate buffer, pH 7.0) and by mechanical removal with a rubber policeman. The membrane was dried and fixed in methanol for 1 min. Afterwards the nuclei were stained with hemacolor (Merck), washed twice with Weise buffer and embedded on a microscope slide coated with immersion oil. The number of invasive tumor cells was evaluated by a microscopic test raster ocular (Zeiss, 400-fold magnification). For a single determination, ten different views per well with a combined membrane surface of 2.5 mm^2^ were evaluated. For statistical confirmation, a mean value and a standard error were calculated from the results [47].

### Analysis of cell proliferation

To study the effect of extracellular calcium on proliferation of primary RCC cells, a colorimetric BrdU incorporation assay (Roche, Mannheim, Germany) was performed. The cells were seeded into a 96 well plate (5 × 10^3^ cells/well), cultured for 48 h and treated in quadruplicate by distinct calcium concentrations (0 mM, 2.5 mM, 5 mM, 10 mM) for 30 min. The CaSR specificity of the observed effect was analyzed by pretreating the cells with NPS 2143 (5 μM) for 1 h (first 30 min without, then 30 min together with calcium). BrdU solution (10 μM) was added to the cells without replacing the NPS 2143 and/or calcium containing culture medium and incubated for 2 h in presence of calcium at 37°C in a humidified atmosphere containing 5% CO_2_ in air. The tumor cells were fixed and the DNA was denatured in one step by adding fixDenat solution for 30 min. Incorporated BrdU was detected by an anti-BrdU-POD antibody within 60 min. The immune complex was detected by a subsequent substrate reaction and quantified by measuring the absorbance at 450 nm (reference wavelength 690 nm) [48].

### Human phospho-kinase array

The activity of 46 intracellular signaling kinases was quantified by using a human phospho-kinase array (R&D, Minneapolis, USA). The kinase array was performed in accordance with the instructions in the manual. Briefly, protein extracts from primary renal tumor cells (cultivated from patients developing no or bone metastases) were prepared by using 200 μl lysis buffer 6 included in the kit. The cells were rinsed twice with ice-cold PBS and scraped off with a rubber policeman in lysis buffer. After 30 min incubation on ice, the extracts were centrifuged at 14.000 rpm, 4°C for 10 min. Protein concentrations were determined using BCA (bicinchoninic acid) reagent (Thermo Scientific, Rockford, USA). The phospho-kinase array membranes were incubated with array buffer 1 for 1 h on a rocking platform. On each membrane 1 ml of the protein lysates (300 μg) were added and incubated overnight at 4°C on a rocking platform. The membranes were washed three times with washing buffer and shaken with antibody cocktails for 2 h. After a 30-minute treatment with streptavidin-HRP solution, the membranes were exposed to a chemiluminescent reagent. Positive signals were visualized using a Chemiluminescence-Imaging System. The amount of protein in each spot was calculated by using Image J software (NIH).

### Western blot analysis

For preparation of protein extracts, renal tumor and normal tissue was pulverized with a mortar under liquid nitrogen and suspended on ice in lysis buffer (20 mM Hepes, pH 7.7, 0.2 M NaCl, 1.5 mM MgCl_2_, 0.4 mM EDTA, 1% Triton X-100, 0.5 mM DTT including protease and phosphatase inhibitors (Sigma, Steinheim, Germany)). For preparation of protein extracts from cell culture, the cells were rinsed twice with ice-cold phosphate-buffered saline (PBS) and scraped off with a rubber policeman in lysis buffer. After 30 min incubation on ice the extracts were centrifuged at 14.000 rpm, 4°C for 10 min. Protein concentrations of the extracts were determined using BCA (bicinchoninic acid) reagent (Thermo Scientific, Rockford, USA). Equal amounts of protein extracts (100 μg per lane) were separated by SDS-PAGE (sodium dodecyl sulfate-polyacrylamide) gel electrophoresis of 10% polyacrylamide (Rotiphorese-Gel 30, Roth, Karlsruhe, Germany) and transferred onto polyvinylidene fluoride membranes (Poly Screen PVDF Transfer Membrane, Perkin Elmer, Rodgau, Germany) by semi-dry blotting. The membrane was blocked in Roti-block blocking solution (Roth, Karlsruhe, Germany) for 1 h. The primary antibodies were incubated in blocking solution (tris buffered saline (TBS), 0.1% Tween 20 and 5% non-fat milk) at 4°C overnight. The antibodies against CaSR (Sigma, Steinheim, Germany), PTEN (phosphatase and tensin homolog deleted on chromosome ten) (monoclonal, rabbit, Cell Signaling), phospho-AKT (Ser473, Thr308, Cell Signaling, Danvers, USA), phospho-ERK (Thr202/Tyr204, Tyr185/Tyr187, Cell Signaling, Danvers, USA) were diluted 1:1000, anti-β-actin (Sigma Aldrich, Taufkirchen, Germany) was diluted 1:5000. The horseradish peroxidase-conjugated secondary antibody (rabbit anti-mouse and goat anti-rabbit, Dako, Hamburg, Germany) was incubated for 1 h at room temperature. Antigens were visualized by an enhanced chemiluminescence solution (ECL, Perkin Elmer Life Sciences, Waltham, USA) using a Chemiluminescence-Imaging System (Fusion-SL4.2 MP, Peqlab, Erlangen, Germany). The amount of expressed protein was calculated analogously by computer-aided integration of the band using Image J software (NIH, USA) after subtraction of the background and referred to the value of total protein, quantified by Coomassie staining of the membrane, (for tissue specimens) and β-actin (for cell extracts), respectively [47].

### Statistical analysis

For statistical analyses IBM-SPSS 19.0 software and Excell 2010 was applied. *CaSR* mRNA expression in renal tumor and normal tissue was quantified and presented as relative units. All other results using primary RCC cells were presented in% of the untreated non-metastasizing cells or related to untreated cells. Differences in the expression of CaSR, cell migration and proliferation were performed using the Student’s *T*-test. Differences were considered statistically significant at p < 0.05.

## Abbreviations

AKT: AKT8 virus oncogene cellular homolog; BrdU: Bromodeoxyuridine; CaSR: Calcium-sensing receptor; ERK: Extracellular signal-regulated kinase; JNK: Jun N-terminal kinase; PLC: Phospholipase C; PTEN: Phosphatase and tensin homologue deleted on chromosome 10; RCC: Renal cell carcinoma.

## Competing interests

The authors declare that they have no competing interests.

## Authors’ contributions

EJ, WB, DP, RCR: Substantial contributions to conception and design. EJ, TH: Acquisition of data. EJ, WB: Analysis and interpretation of data. EJ, WB, DP, FCR: Writing, review and/or revision of the manuscript. WB, KJ, CH, FCR, JWT: Final approval of the version to be published. WB, FCR: Study supervision. All authors read and approved the final manuscript.
